# Dipotassium 4,4′-(hexane-3,4-di­yl)bis­(benzene­sulfonate) dihydrate

**DOI:** 10.1107/S160053680802120X

**Published:** 2008-07-16

**Authors:** Liana Orola, Mikelis V. Veidis, Sergey Belyakov, Andris Actins

**Affiliations:** a University of Latvia, Kr. Valdemara 48, Riga, LV 1013, Latvia; b Latvian Institute of Organic Synthesis, Aizkraukles 21, Riga, LV 1006, Latvia

## Abstract

The anion of the title compound, also called sygethin dihydrate, 2K^+^·C_18_H_20_O_6_S_2_^2−^·2H_2_O, has crystallographic inversion symmetry. The K^+^ cation is surrounded by eight O atoms in a distorted cubic coordination geometry, forming extended K—O—S networks. There are also O—H⋯O hydrogen bonds.

## Related literature

For the synthesis, see: Torf & Khromov-Borisov (1961 ▶). For general background, see: Svergun (1979 ▶). For a related structure, see: Weeks *et al.* (1973 ▶).
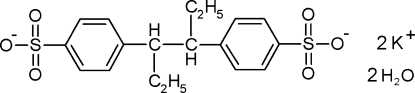


## Experimental

### 

#### Crystal data

2K^+^·C_18_H_20_O_6_S_2_^2−^·2H_2_O
*M* *_r_* = 255.36
Triclinic, 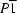

*a* = 5.8741 (5) Å
*b* = 6.5684 (5) Å
*c* = 15.2335 (14) Å
α = 84.272 (4)°
β = 83.768 (5)°
γ = 76.522 (6)°
*V* = 566.51 (8) Å^3^
*Z* = 1
Mo *K*α radiation
μ = 0.64 mm^−1^
*T* = 298 K
0.27 × 0.19 × 0.14 mm

#### Data collection

Nonius KappaCCD diffractometer
Absorption correction: none
4330 measured reflections
2576 independent reflections
1918 reflections with *I* > 2σ(*I*)
*R*_int_ = 0.027

#### Refinement

*R*[*F* ^2^ > 2σ(*F* ^2^)] = 0.051
*wR*(*F* ^2^) = 0.122
*S* = 0.89
1918 reflections
137 parameters
H-atom parameters constrained
Δρ_max_ = 1.49 e Å^−3^
Δρ_min_ = −0.42 e Å^−3^

### 

Data collection: *COLLECT* (Nonius, 2001 ▶); cell refinement: *DENZO*/*SCALEPACK* (Otwinowski & Minor, 1997 ▶); data reduction: *DENZO*/*SCALEPACK*; program(s) used to solve structure: *SIR92* (Altomare *et al.*, 1994 ▶); program(s) used to refine structure: *CRYSTALS* (Betteridge *et al.*, 2003 ▶); molecular graphics: *ORTEP-3 for Windows* (Farrugia, 1997 ▶); software used to prepare material for publication: *CRYSTALS*.

## Supplementary Material

Crystal structure: contains datablocks global, I. DOI: 10.1107/S160053680802120X/cf2208sup1.cif

Structure factors: contains datablocks I. DOI: 10.1107/S160053680802120X/cf2208Isup2.hkl

Additional supplementary materials:  crystallographic information; 3D view; checkCIF report

## Figures and Tables

**Table 1:** Selected bond lengths (Å)

K14—O9^i^	2.733 (3)
K14—O7^ii^	2.736 (3)
K14—O15^ii^	2.816 (3)
K14—O7^iii^	2.834 (3)
K14—O9	2.934 (3)
K14—O15	2.937 (3)
K14—O8^iii^	2.970 (3)
K14—O7	3.211 (3)

Symmetry codes: (i) 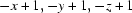; (ii) 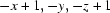; (iii) 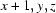.

**Table 2:** Hydrogen-bond geometry (Å, °)

*D*—H⋯*A*	*D*—H	H⋯*A*	*D*⋯*A*	*D*—H⋯*A*
O15—H5⋯O8^iv^	0.84	2.00	2.790 (2)	156

Symmetry code: (iv) 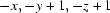.

**Fig. 1.**
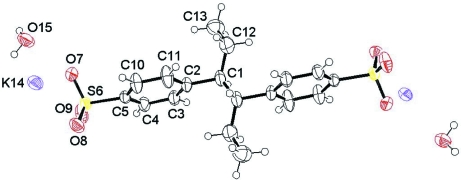
The structure of the title compound, with displacement ellipsoids at the 50% probability level. [Symmetry code for unlabeled atoms: 1-x, -y, -z.]

**Fig. 2.**
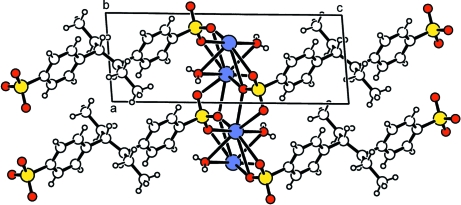
Packing diagram of the title compound.

## supplementary crystallographic information

### Comment

The synthesis has been described by Torf & Khromov-Borisov (1961). Replacement of the two OH groups of the estrogen hexestrol molecule with two KSO_3_ groups results in the formation of the dipotassium salt of 4,4'-(1,2-diethyl-1,2-ethanediyl)bis(benzenesulfonic acid), also known as sygethin. Although the placement of carbon atoms in sygethin is very similar to hexestrol (Weeks *et al.*, 1973) sygethin does not show estrogen-type activity (Svergun, 1979).

The crystal structure of the title compound has been determined. Fig. 1 illustrates the structure. The anion is located on a center of symmetry. The unit cell contains one sygethin anion, two potassium cations and two water molecules.

The packing diagram, Fig. 2, indicates that there are eight oxygen atoms coordinated to the potassium ion in a very distorted cubic geometry: six oxygen atoms are from four sygethin SO_3_ ions and two oxygen atoms are from the two water molecules. A hydrogen bond is formed by each water molecule and sygethin.

### Experimental

The title compound was supplied by Grindeks Company. To grow crystals suitable for single-crystal diffraction study, the powder form of sygethin dihydrate was dissolved in water at 333 K to obtain a saturated solution. After filtration, the saturated solution was diluted with approximately 50% more water and allowed to crystallize in a petri dish at ambient temperature.

### Refinement

The hydrogen atoms were all located in a difference Fourier map. Hydrogen atoms attached to carbon atoms were repositioned goemetrically. During refinement, hydrogen atoms were constrained to the riding mode. *U*_iso_(H)=*xU*_eq_(C,*O*), where the average values of *x* are 1.66 for H atoms of the methyl group, 1.2 to 1.29 for H atoms attached to the remaining C atom, and 1.41 for the H atoms of the water molecule.

### Crystal data

**Table:**

2K^+^·C_18_H_20_O_6_S_2_^2–^·2H_2_O	*Z* = 1
*M**_r_* = 255.36	*F*_000_ = 266
Triclinic, *P*1	*D*_x_ = 1.497 Mg m^−^^3^*D*_m_ = Mg m^−^^3^*D*_m_ measured by ?
Hall symbol: -P 1	Mo *K*α radiation λ = 0.71073 Å
*a* = 5.8741 (5) Å	Cell parameters from 2576 reflections
*b* = 6.5684 (5) Å	θ = 1.4–27.4º
*c* = 15.2335 (14) Å	µ = 0.64 mm^−^^1^
α = 84.272 (4)º	*T* = 298 K
β = 83.768 (5)º	Prism, colourless
γ = 76.522 (6)º	0.27 × 0.19 × 0.14 mm
*V* = 566.51 (8) Å^3^	

### Data collection

**Table:**

Nonius KappaCCD diffractometer	1918 reflections with *I* > 2σ(*I*)
Radiation source: Enhance (Mo) X-ray Source	*R*_int_ = 0.027
Monochromator: graphite	θ_max_ = 27.4º
*T* = 298 K	θ_min_ = 1.4º
φ and ω scans	*h* = −7→7
Absorption correction: none	*k* = −8→8
4330 measured reflections	*l* = −18→19
2576 independent reflections	

### Refinement

**Table:**

Refinement on *F*^2^	H-atom parameters constrained
Least-squares matrix: full	*w* = [1-(*F*_o_-*F*_c_)^2^/36σ^2^(*F*)]^2^/[53.8T_0_(x) + 84.3T_1_(x) + 51.6T_2_(x) + 20.0T_3_(x) + 5.48T_4_(x)] where T_i_ are Chebychev polynomials and x = *F*_c_/*F*_max_
*R*[*F*^2^ > 2σ(*F*^2^)] = 0.051	(Δ/σ)_max_ = 0.0003
*wR*(*F*^2^) = 0.122	Δρ_max_ = 1.49 e Å^−^^3^
*S* = 0.89	Δρ_min_ = −0.42 e Å^−^^3^
1918 reflections	Extinction correction: none
137 parameters	

### Fractional atomic coordinates and isotropic or equivalent isotropic displacement parameters (Å^2^)

**Table:**

	*x*	*y*	*z*	*U*_iso_*/*U*_eq_	
C1	0.5804 (7)	−0.0364 (6)	0.0370 (2)	0.0454	
C2	0.4756 (6)	0.0667 (5)	0.1221 (2)	0.0398	
C3	0.5685 (7)	0.2183 (5)	0.1527 (2)	0.0455	
C4	0.4714 (7)	0.3172 (5)	0.2292 (2)	0.0431	
C5	0.2797 (5)	0.2595 (5)	0.27676 (19)	0.0324	
S6	0.15644 (14)	0.37197 (12)	0.37713 (5)	0.0329	
O7	0.1593 (5)	0.1938 (4)	0.44237 (15)	0.0503	
O8	−0.0830 (4)	0.4813 (4)	0.36269 (17)	0.0476	
O9	0.3068 (5)	0.5041 (5)	0.39615 (18)	0.0554	
C10	0.1865 (7)	0.1054 (7)	0.2483 (2)	0.0490	
C11	0.2858 (7)	0.0092 (8)	0.1721 (3)	0.0564	
C12	0.6723 (9)	−0.2695 (7)	0.0533 (3)	0.0589	
C13	0.8749 (8)	−0.3309 (7)	0.1140 (3)	0.0603	
K14	0.67561 (14)	0.20792 (11)	0.48854 (5)	0.0427	
O15	0.3244 (5)	0.1011 (4)	0.62693 (19)	0.0594	
H11	0.7195	0.0235	0.0172	0.0592*	
H31	0.7003	0.2542	0.1201	0.0587*	
H41	0.5361	0.4240	0.2493	0.0543*	
H101	0.0552	0.0653	0.2817	0.0628*	
H111	0.2199	−0.0957	0.1524	0.0748*	
H121	0.7280	−0.3309	−0.0033	0.0684*	
H122	0.5410	−0.3272	0.0825	0.0689*	
H131	0.9207	−0.4839	0.1192	0.0894*	
H132	1.0067	−0.2743	0.0867	0.0891*	
H133	0.8213	−0.2757	0.1713	0.0894*	
H5	0.2249	0.2110	0.6406	0.0828*	
H13	0.4165	0.0717	0.6665	0.0829*	

### Atomic displacement parameters (Å^2^)

**Table:**

	*U*^11^	*U*^22^	*U*^33^	*U*^12^	*U*^13^	*U*^23^
C1	0.053 (2)	0.050 (2)	0.0306 (16)	−0.0163 (14)	0.0018 (14)	−0.0036 (16)
C2	0.0498 (19)	0.0424 (18)	0.0251 (14)	−0.0110 (13)	−0.0015 (12)	−0.0038 (15)
C3	0.066 (2)	0.0375 (17)	0.0340 (16)	−0.0099 (13)	0.0122 (15)	−0.0186 (17)
C4	0.062 (2)	0.0350 (16)	0.0350 (16)	−0.0118 (13)	0.0071 (15)	−0.0172 (16)
C5	0.0360 (15)	0.0342 (15)	0.0247 (13)	−0.0073 (11)	−0.0044 (11)	−0.0006 (13)
S6	0.0364 (4)	0.0328 (4)	0.0272 (4)	−0.0097 (3)	−0.0024 (3)	0.0000 (3)
O7	0.0666 (17)	0.0453 (14)	0.0291 (12)	−0.0015 (10)	0.0008 (11)	0.0045 (13)
O8	0.0413 (14)	0.0446 (14)	0.0498 (14)	−0.0112 (11)	−0.0066 (11)	0.0089 (11)
O9	0.0584 (17)	0.0668 (18)	0.0492 (15)	−0.0335 (13)	0.0058 (12)	−0.0235 (14)
C10	0.0441 (19)	0.068 (2)	0.0424 (18)	−0.0273 (17)	0.0078 (15)	−0.0226 (18)
C11	0.053 (2)	0.080 (3)	0.049 (2)	−0.039 (2)	0.0069 (16)	−0.032 (2)
C12	0.073 (3)	0.055 (2)	0.049 (2)	−0.0125 (18)	0.0002 (19)	−0.014 (2)
C13	0.062 (3)	0.054 (2)	0.066 (3)	−0.005 (2)	−0.016 (2)	−0.010 (2)
K14	0.0488 (5)	0.0328 (4)	0.0463 (4)	−0.0084 (3)	−0.0041 (3)	−0.0066 (3)
O15	0.0713 (19)	0.0425 (14)	0.0553 (16)	−0.0111 (12)	−0.0175 (14)	0.0138 (13)

### Geometric parameters (Å, °)

**Table:**

C1—C1^i^	1.518 (7)	C11—H111	0.952
C1—C2	1.525 (4)	C12—C13	1.542 (6)
C1—C12	1.505 (6)	C12—H121	0.980
C1—H11	0.993	C12—H122	0.982
C2—C3	1.382 (5)	C13—H131	0.975
C2—C11	1.386 (5)	C13—H132	0.972
C3—C4	1.393 (4)	C13—H133	0.971
C3—H31	0.938	O15—H5	0.844
C4—C5	1.380 (5)	O15—H13	0.832
C4—H41	0.961	K14—O9^ii^	2.733 (3)
C5—S6	1.773 (3)	K14—O7^iii^	2.736 (3)
C5—C10	1.382 (5)	K14—O15^iii^	2.816 (3)
S6—O7	1.456 (3)	K14—O7^iv^	2.834 (3)
S6—O8	1.452 (2)	K14—O9	2.934 (3)
S6—O9	1.442 (3)	K14—O15	2.937 (3)
C10—C11	1.383 (5)	K14—O8^iv^	2.970 (3)
C10—H101	0.952	K14—O7	3.211 (3)
			
C1^i^—C1—C2	111.4 (4)	O7—S6—O9	112.47 (18)
C1^i^—C1—C12	116.9 (4)	O8—S6—O9	114.94 (17)
C2—C1—C12	112.1 (3)	C5—C10—C11	119.9 (3)
C1^i^—C1—H11	104.1	C5—C10—H101	119.6
C2—C1—H11	104.9	C11—C10—H101	120.5
C12—C1—H11	106.2	C2—C11—C10	121.4 (3)
C1—C2—C3	121.3 (3)	C2—C11—H111	119.0
C1—C2—C11	121.1 (3)	C10—C11—H111	119.6
C3—C2—C11	117.6 (3)	C1—C12—C13	114.2 (4)
C2—C3—C4	121.9 (3)	C1—C12—H121	109.5
C2—C3—H31	117.9	C13—C12—H121	108.1
C4—C3—H31	120.2	C1—C12—H122	106.9
C3—C4—C5	119.1 (3)	C13—C12—H122	107.6
C3—C4—H41	121.1	H121—C12—H122	110.5
C5—C4—H41	119.8	C12—C13—H131	106.9
C4—C5—S6	121.1 (2)	C12—C13—H132	109.2
C4—C5—C10	120.0 (3)	H131—C13—H132	109.6
S6—C5—C10	118.9 (3)	C12—C13—H133	109.4
C5—S6—O7	104.86 (14)	H131—C13—H133	111.4
C5—S6—O8	106.39 (14)	H132—C13—H133	110.4
O7—S6—O8	110.82 (17)	H5—O15—H13	106.5
C5—S6—O9	106.55 (15)		

Symmetry codes: (i) −*x*+1, −*y*, −*z*; (ii) −*x*+1, −*y*+1, −*z*+1; (iii) −*x*+1, −*y*, −*z*+1; (iv) *x*+1, *y*, *z*.

### Hydrogen-bond geometry (Å, °)

**Table:**

*D*—H···*A*	*D*—H	H···*A*	*D*···*A*	*D*—H···*A*
O15—H5···O8^v^	0.84	2.00	2.790 (2)	156

Symmetry codes: (v) −*x*, −*y*+1, −*z*+1.
